# Palm Lipid Emulsion Droplet Crystallinity and Gastric Acid Stability in Relation to *in vitro* Bioaccessibility and *in vivo* Gastric Emptying

**DOI:** 10.3389/fnut.2022.940045

**Published:** 2022-07-22

**Authors:** Samar Hamad, Run Chen, Zhitong Zhou, Pedram Nasr, Ye Ling Li, Niloufar Rafiee Tari, Michael A. Rogers, Amanda J. Wright

**Affiliations:** ^1^Department of Human Health and Nutritional Sciences, University of Guelph, Guelph, ON, Canada; ^2^Department of Food Science, University of Guelph, Guelph, ON, Canada

**Keywords:** emulsion, lipid bioaccessibility, triacylglycerol, gastric emptying, *in vitro* digestion, TIM-1, human study correlation

## Abstract

It is poorly understood how the physical state of emulsified triacylglycerol (TAG) alters colloidal behavior in the gastrointestinal tract to modulate lipid digestion and absorption. We, therefore, aimed to investigate the individual and combined effects on fatty acid (FA) bioaccessibility using the dynamic TIM-1 *in vitro* digestion model and integrate the results with those from a human clinical study. Four 20% oil-in-water emulsions with overlapping particle size distributions contained either partially crystalline solid (palm stearin) or liquid (palm olein) lipid droplets at 37°C and either the colloidally acid-stable Tween 80 (2.2%) or acid-unstable Span 60 (2.5%) emulsifier. Experimental meals were fed to the TIM-1, and jejunal and ileal dialysates were analyzed over 6 h to measure free FA concentration. Cumulative FA bioaccessibility was significantly higher for the liquid stable emulsion compared to all others (*p* < 0.05), which did not differ (*p* > 0.05). Emulsified TAG physical state was associated with differences in overall bioaccessibility (higher for liquid state TAG) in the colloidally stable emulsions, but this difference was blunted in droplets susceptible to acidic flocculation. In contrast, human postprandial TAG concentrations did not differ significantly between the emulsions. The discrepancy may relate to differences in *in vivo* gastric emptying (GE) as evidenced by ultrasonography. When the *in vivo* differences in GE were accounted for in follow-up TIM-1 experiments, the findings aligned more closely. Cumulative FA bioaccessibility for the liquid stable emulsion no longer differed significantly from the other emulsions, and SU’s bioaccessibility was the lowest, consistent with the *in vivo* observations. This work highlights the potential for TAG physical state and colloidal stability to interactively alter behavior in the gastrointestinal tract with implications for FA absorption, and the importance of establishing and improving *in vitro–in vivo* correlations in food-nutrition research.

## Introduction

Excess energy intake, including dietary fat, is a leading cause of obesity (1) and a risk factor for many diseases, including cardiovascular diseases (CVD), type 2 diabetes, and some cancers (2–4). Postprandial lipemia (PPL) is recognized as a CVD risk factor (5), and lipid absorption correlates with fat-soluble vitamin absorption (6) and, more recently, endotoxemia (7). Therefore, there may be advantages to modulating lipid bioavailability (8), i.e., to promote or delay it or to change the overall absorption (9, 10). Research into strategies to structure lipid-rich foods, including emulsions, that resist or enhance digestion, altering metabolism is rationalized. These include emulsification, emulsion droplet size, interfacial properties, physical lipid state, and physicochemical properties of the surrounding food matrix (11–13). Understanding the specific influence of triacylglycerol (TAG) physical state and solid fat content (SFC) at physiological temperature, i.e., 37°C is important for designing foods that capitalize on the dynamics in the digestive tract to tailor PPL and lipid bioavailability.

*In vitro* digestion methods have yielded interesting insights in this area. Crystalline TAGs induce partial coalescence and different gastric structures that have been associated with attenuated *in vitro* lipolysis (14). Also, *in vitro* digestibility was higher for liquid canola-oil-based emulsions stabilized with non-ionic emulsifiers (Tween 20 and Poloxamer 188) than for high SFC emulsions with fully hydrogenated canola stearin (15). In addition, *in vitro* digestion kinetics were inversely associated with SFC for whey protein stabilized oil-in-water emulsions based on blends of soybean oil and fully hydrogenated soybean oil (13). Tempering has also been used to achieve differing SFCs for emulsions with identical compositions, thereby isolating the role of TAG physical state without confounding differences in fatty acid (FA) composition (16–19). The rate and extent of lipolysis increased with SFC and remained constant for undercooled emulsions stabilized with Tween 80 using palm stearin and palm olein blends (16). *In vitro* lipolysis of partially crystallized tripalmitin (and SDS) (17, 18) and palm stearin (and Span 60) (19) emulsions showed attenuated FA bioaccessibility compared to their undercooled counterparts. Similar differences were observed following consumption of the tempered palm stearin emulsions in a human study comparing PPL (20). Moreover, short-term appetite suppression was enhanced for the undercooled versus partially crystalline palm stearin emulsion (21). Aside from TAG crystallinity, the ingested emulsions were identical, and yet the droplets containing solid fat destabilized more extensively during exposure to simulated gastric conditions (19). Collectively, these studies highlight the important roles of TAG crystallinity and emulsion acid stability in determining lipid digestion and postprandial metabolism, but questions about their combined effects remain.

Emulsification in the gastrointestinal (GI) tract occurs *via* mechanical peristaltic forces and the addition of surface-active molecules that changes the interfacial area available for lipolysis (12). As an example, protein emulsifiers are digested by pepsin, whereas ionic emulsifiers experience different degrees of electrostatic repulsion depending on the gastric and luminal pH, leading to differences in emulsion stability, droplet flocculation, and potentially coalescence and phase separation (22, 23). These effects can translate into altered lipid digestion profiles. For example, when olive oil emulsions with sucrose-ester surfactants were exposed to simulated gastric conditions, droplet size increased, corresponding to lower lipolysis than emulsions stabilized with Tween 80 (24). Human-level comparisons between acid-stable (Tween 60) versus acid-unstable (Span 60) emulsions indicate that gastric emptying (GE), cholecystokinin release, gallbladder contraction, PPL, and satiety all change with emulsion colloidal stability (25–27). MRI imaging revealed emulsion phase separation along with faster GE, delayed plasma lipid response, and lower satiety when participants consumed an acid-unstable palmitate-enriched olive oil-in-water emulsion (emulsified with Span 60) compared with an acid-stable emulsion (emulsified with Tween 60) (25). Steingoetter et al. showed that emulsifier acid stability, combined with solid fat to prevent emulsion redispersal, resulted in lower lipid bioavailability of oil-in-water emulsions (28), but without a treatment containing solid fat that was acid-stable, conclusions based on lipid physical state alone could not be drawn. The aim herein was to investigate the independent and combined effects of TAG crystallinity and emulsifier acid stability on TAG lipolysis and FA bioaccessibility using static and dynamic *in vitro* digestion methods, respectively, to compare four comprehensively characterized 20% oil-in-water emulsions containing either palm stearin (partially solid at 37°C) or palm olein (liquid at 37°C), and either Tween 80 (acid-stable emulsifier) or Span 60 (acid-unstable emulsifier). These emulsions were previously consumed by participants in a randomized crossover study in which different gastric microstructures were observed directly by ultrasonography and associated with different rates of GE (29).

*In vitro* digestion models allow gaining mechanistic insights into how food structures change in the gastrointestinal tract to differentially affect postprandial metabolism. The dynamic TIM-1 multicompartment system simulates the luminal conditions of the upper GI tract from the stomach, duodenum, and jejunum to the ileum, integrating physiological factors including meal transit time, peristaltic contractions, enzyme concentration, and pH changes (30–32). Dialysis membranes fractionate hydrolyzed and solubilized free fatty acids (FFA) from the luminal fluid (33) as a measure of bioaccessibility. Although TIM-1 results have correlated well with *in vivo* results (34–36), there are relatively few published reports and these exist for a relatively limited range of foods, especially in scenarios where the meals could have substantially different GE behaviors. Therefore, the secondary aim of this work was to compare the TIM-1 *in vitro* findings to those from an *in vivo* PPL study where healthy adults consumed the same four emulsions (29) in order to explore and improve the predictive value of *in vitro* digestion methods, addressing a recognized need in the food-nutrition community (37).

## Materials and Methods

### Materials

Palm stearin (Bunge Oils Inc., Bradley, IL, United States), palm oil olein—organic (#S1385, Jedwards International, Inc., Braintree, MA, United States), sorbitan monostearate (Span 60, Croda Canada Ltd.), polyoxyethylene sorbitan monooleate (Tween 80, Croda Canada Ltd.), and deionized water were used to prepare the emulsions. We used the following reagents from Sigma-Aldrich (St. Louis, MO, United States) for the TIM-1 experiments: lipase from porcine pancreas (type II, 100–500 U/mg protein), pepsin from porcine gastric mucosa (≥2,500 U/mg protein), α-amylase from *Bacillus* sp. (type II-A, ≥1,500 U/mg protein), porcine bile extract [95% (50% cholic acid sodium salt and 50% deoxycholic acid sodium salt)], bovine pancreas trypsin powder [≥7,500 N-α-benzoyl-L-arginine ethyl ester (BAEE) U/mg], anhydrous ethanol, HCl (37%), NaHCO_3_ (≥99%), hydroxypropylmethyl cellulose powder (HPMC), NaCl (≥99%), KCl (≥99%), and CaCl_2_ (≥96%). Fresh porcine bile was collected from Conestoga Meat Packers (Breslau, ON, Canada) at the time of slaughter, pooled and filtered (cheesecloth) into allocated single-use portions and stored at −30°C.

### Emulsion Preparation

The study emulsions consisted of LS (liquid lipid-acid stable emulsifier), LU (liquid lipid-acid unstable emulsifier), SS (solid lipid-acid stable emulsifier), and SU (solid lipid-acid unstable emulsifier). To determine the effects of droplet TAG crystallinity, they were formulated with palm stearin (partially crystalline at 37°C) or palm olein (liquid at 37°C). They were also formulated with either Tween 80 or Span 60 to be either colloidally stable or unstable, respectively, in the presence of gastric acidity. The emulsifier concentrations were selected to achieve overlapping particle size distributions (PSDs) based on preliminary experiments. Span 60 is a relatively low HLB value (4.7) emulsifier selected based on previous observations that monomodal PSDs could be obtained for undercooled and maximally crystallized palm stearin emulsions at concentrations permissible for consumption in the corresponding human studies (19). The total weight of the emulsions was 300 g. First, 60 g of either palm olein or stearin, with either 6.6 g Tween 80 or 7.5 g Span 60, was melted together in a glass jar (oven temperature at 85°C for 30 min to remove any lipid crystal memory), followed by the addition of 233 g boiling deionized water. A coarse emulsion was achieved using an ULTRA TURRAX^®^ (IKA^®^ T18 basic, Staufen, Germany) at 12,000 min^–1^ for 2 min and then passed through a microfluidizer (M-110EH, Microfluidics, Westwood, MA, United States) three times at 125 MPa with the machine’s piping immersed in a water reservoir at ∼95°C, using portable immersion heaters. The resulting fine emulsion with palm olein (LS and LU) was held at room temperature (21 ± 1°C), and those with palm stearin (SS and SU) were placed in a pre-cooled jar, submerged in ice water for 20 min, and then refrigerated (5°C) for at least 18 h to induce crystallization of the lipid droplets. Before all analyses and experiments, the emulsions were warmed to 37°C for 1 h. All emulsions were stored and used within 7 days after confirming that PSDs and melting behavior remained consistent over this period (data not shown).

### Physical Characterization of Emulsions

The FA composition of the bulk lipids and emulsifiers was determined by gas chromatography (Agilent 7890A GC) with flame ionization detection (Agilent Technologies Inc., DE, United States) (35) after extracting the total lipids using the Folch method (38). Transesterification using 14% boron trifluoride in methanol and re-suspending in hexane was performed before analysis. Laser diffraction [Mastersizer 2000S (Malvern Instruments Inc., Southborough, MA, United States)] determined the PSDs, volume-weighted, D_4,3,_ and surface-weighted, D_3,2_, and mean diameters using refractive indices of 1.45 and 1.33 for the lipid and water phases, respectively. The emulsion droplet surface charge (ζ-potential) was measured by a particle electrophoresis instrument (Zetasizer Nano ZS, Malvern Instruments Inc., Southborough, MA, United States). Emulsion SFC was measured at 37°C according to AOCS official method Cd 16b-93 using a Bruker Minispec PC/20 series pulsed nuclear-magnetic-resonance spectrophotometer (Bruker Spectrospin, Milton, ON, Canada). A differential scanning calorimeter (Q2000 model, TA Instruments, Mississauga, ON, Canada) determined the melting behavior of each emulsion, as previously described (19), by heating the samples from 37 to 85°C at 5°C/min. The apparent viscosity of each emulsion was determined as a function of the shear rate before and after 1 h of static gastric digestion, using a controlled strain rheometer (MCR 301, Anton Paar GmbH, Ostfildern, Germany), equipped with a concentric cylinder geometry (CC 27, 27 mm diameter), and a Peltier temperature controller to maintain 37°C.

### Exposure to Static *in vitro* Gastric Digestion Conditions

Static *in vitro* digestion experiments confirmed and compared the colloidal behavior of each emulsion during exposure to simulated gastric digestion fluid (SGF) as described previously (30). Five milliliters of each emulsion and 5 ml of SGF, containing 2,000 U/mL pepsin and 12.6 mg/mL of the antioxidant pyrogallol, were added to amber glass jars with four 10-mm glass beads and incubated in a shaking water bath [37°C, 250 rpm (New Brunswick Scientific Co. Inc., NJ, United States)] for 2 h at pH 3. After digestion, samples were immediately analyzed for PSD, using the Mastersizer as described above. For SU and LU, the pH was adjusted to 7.0 by adding 1.0 N NaOH to assess re-dispersion (visually) and PSD.

### Determination of Lipid Bioaccessibility

The TNO *in vitro* digestion upper GI model-1 TIM-1 (Zeist, Netherlands) dynamically simulated digestive conditions and was used to investigate the role of droplet SFC and colloidal stability on lipid bioaccessibility, the precursor to lipid absorption. According to the fed-state protocol (39), TIM-1 secretions and start residues consisted of 1 M HCL, gastric enzyme solution, 1 M sodium bicarbonate, hydroxypropylmethyl cellulose (HPMC), 7% pancreatin solution, small intestinal electrolyte solution (SIES 1×), fresh bile, ileal secretion, and jejunal secretion. The 7% pancreatin solution, prepared by mixing 237 ± 3 g water and 35 ± 0.2 g pancreatic powder with magnetic stirring at room temperature for 10 min, was centrifuged for 20 min (12,500 × *g* at 4°C), and the supernatant was collected. Gastric electrolyte solution (GES 1×) was prepared with 53 ± 0.5 g GES 10× and 450 ± 5 g water, and gastric enzyme solution by mixing (by magnetic stirring for 10 min at room temperature) 249 ± 3 g GES 1×, 2.5 ± 0.1 ml 1 M sodium acetate buffer (pH = 5), 167 mg lipase, 480 mg pepsin, and 13.5 mg amylase and stored at 0–5°C. The small intestine electrolyte solution was prepared from 88 ± 1 g SIES 25× and 1920 ± 10 g water. Bile was warmed to 37°C in a 60°C water bath before use. Jejunal secretions were prepared by mixing 88 ± 1 g SIES 25× and 1,720 ± 10 g water followed by 206 ± 2 g of bile, and ileal secretions were 1,606 g of SIES 1×. Gastric start solution [5 ± 0.1 g gastric enzyme solution (0.40% HPMC and 0.04% bile powder)], duodenal start solution (15 ± 0.3 g SIES 1×, 15 ± 0.3 g 7% pancreatin solution, 30 ± 0.5 g bile, and 2 mg trypsin solution), jejunal start residue solution (35 ± 0.5 g SIES 1×, 35 ± 0.5 g pancreatin solution and 70 ± 1 g bile), and ileal start residue solution (140 ± 1 g SIES 1×) were added directly into the compartments. The “feed meal” consisted of 100 g emulsion, 90 g GES 1×, 50 g water, and 11 mg amylase and was prepared in a beaker, maintained at 37 ± 1°C. The meal and the 50 g rinse water were added to the stomach compartment already conditioned with 10 g GES. Semi-permeable, 0.05-μm pore size capillary membranes (Spectrum Milikros modules M80S-300-01P, Repligen, Waltham, MA, United States) attached at the jejunal and ileal compartments collected the dialysates (i.e., bioaccessible lipids) at 30, 60, 90, 120, 180, 240, 300, and 360 min. These were weighed and sampled into 1.5-ml Eppendorf tubes and stored at −20°C until FFA analysis.

### Quantification of Free Fatty Acids

All dialysate samples were thawed at room temperature, and lipids were extracted by adding 50 μL of sample to an Eppendorf tube with 50 μL 1 M HCL and 500 μL hexane and centrifuged at 14,000 RPM for 30 min (5418 Laboratory Centrifuge, Eppendorf, Hamburg, Germany). The double-layered supernatant was added to 500 μL of hexane, and the tube was rinsed with another 500 μL of hexane. Non-esterified fatty acids (NEFA) were quantified in 96-well plates in duplicate for each TIM-1 run (performed in triplicate) according to the manufacturer instructions [Wako HR series NEFA-HR (2), FUJIFILM Wako Diagnostics, VA, United States] using an enzymatic colorimetric method assay. The cumulative FFA bioaccessibility (%) was the total accumulated lipid bioaccessibility at each time point (e.g., cumulative bioaccessibility at 60 min calculated as the sum of bioaccessibility from 0 to 30 and 30 to 60 min).

### Adjustment of TIM-1 Gastric Emptying Rate

Additional TIM-1 digestions were performed by modifying the fed-state lipid protocol by adjusting the GE rates to those observed for the same emulsions in our previous human study. As previously reported, gastric ultrasound was used to visualize and measure the gastric antrum cross-sectional area at baseline and postprandially (29) from which we quantified GE. Specifically, the percentage GE volume after consuming those same four emulsions was determined at 0, 30, 60, 90, 120, 150, 180, 240, 300, and 360 min, converted to the fraction of meal remaining in the stomach, and used to determine the GE half time *T*_1/2_ using Eq. 1 (40):


(1) $$F=100\times 2^{-{\left(\frac{T}{T_{1/2}}\right)}^{\beta }}$$

where *F* is the meal fraction remaining in the stomach, *T*_1/2_ is the GE half time, and β is a parameter defining the shape of the curve. A total of 80 min is the standard TIM-1 GE *T*_1/2_ used in the initial experiments. The *in vivo* (40) GE *T*_1/2_ ratio observed for LS:SU was 1.55 (i.e., 233 and 150 min for LS and SU, respectively). Thus, the LS emulsion was subsequently digested in the TIM-1 using an adjusted GE *T*_1/2_ of 124 min.

### Data and Statistical Analysis

All experiments were performed in at least triplicate, with analytical duplicates, using samples prepared within 7 days. Microsoft Excel (Redmond, DC, United States) was used to plot the FFA standard curve and to calculate cumulative % bioaccessibility for the TIM-1. GraphPad Prism 8 (San Diego, CA, United States) was used for all calculations and statistical analysis. Area under the curve (AUC) values were calculated using the trapezoid rule. A shifted logistic equation fits the cumulative FA bioaccessibility using GraphPad Prism 8 (San Diego, CA, United States) to model the FFA generation after lipolysis over time as follows (39):

$$C\,\left(t\right)=\frac{C_{a\,s\,y\,m\,p}}{1+e^{\left[k\,\left(t_{c}-t\right)\right]}}-\frac{C_{a\,s\,y\,m\,p}}{1+e^{k\,t_{c}}}$$

where *C*_*asymp*_ is the asymptotic plateau representing the maximum bioaccessible FFAs, *k* is the rate constant of FFA release per unit time (i.e., rate of lipolysis), and *t*_*c*_ is the induction time (i.e., the time to reach effective lipolysis where half the total amount of FFAs are released) (39). Initial values were *C*_*asymp*_ = 40; *k* = 0.00; and *t*_*c*_ = 0.1, followed by the software performing 1,000 iterations.

All time course data were analyzed by two-way repeated-measures ANOVA (RM-ANOVA) with time (seven levels: 0, 30, 60, 90, 120, 180, 240, 300, and 360 min) and treatment (four levels: LS, LU, SS, and SU) as the two factors followed by Bonferroni’s multiple comparison testing when treatment and/or time × treatment interactions were significant. AUC, 6 h % bioaccessibility, rate constant (*k*), and induction time (*t*_*c*_) values were analyzed by one-way ANOVA. Data were normally distributed (passed the Shapiro–Wilk test), there were no missing values, and statistical significance was set at *p* < 0.05. The hourly TIM-1 FFA and AUC values were correlated with the hourly and AUC postprandial TAG response of 15 healthy male adult participants who consumed the same test emulsions across four study visits following an overnight fast, separated by least 6 days (29) using Pearson’s correlation analysis.

## Results

### Emulsion Composition and Physical Properties

**FIGURE 1:**
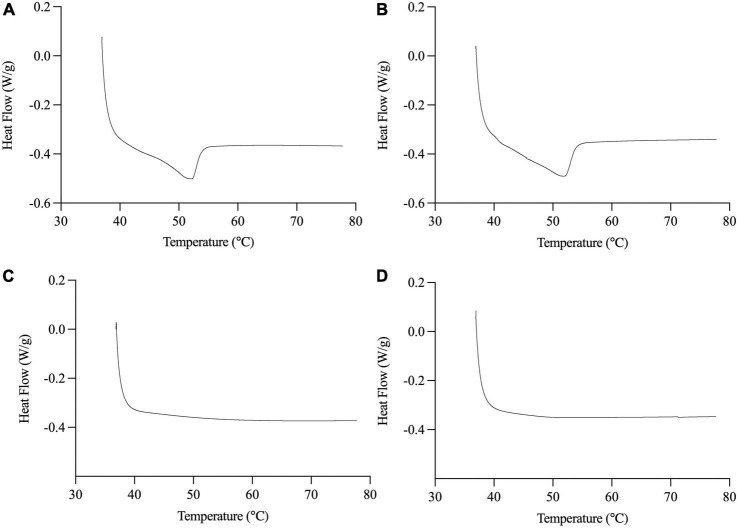
Representative DSC thermograms of melting behavior of SS **(A)**, SU **(B)**, LS **(C)**, and LU **(D)**. Data reported as mean ± SEM, *n* = 3. LS, liquid lipid-acid stable emulsifier; LU, liquid lipid-acid unstable emulsifier; SS, solid lipid-acid stable emulsifier; SU, solid lipid-acid unstable emulsifier.

Compared to palm olein, palm stearin contained significantly more palmitic acid (C16:0) (58.7 ± 0.0 versus 36.4 ± 0.1%) and less oleic acid (C18:1c9) (26.8 ± 0.1 versus 45.2 ± 0.0%) (Table 1), leading to SFC differences between the liquid and solid emulsion droplets (Table 2). The 20% palm stearin oil-in-water emulsions possessed ∼9.3% SFC, representing ∼44–49% of the emulsified TAGs. The emulsions, stabilized with Span 60 which melts at ∼56°C, trended to higher SFC than the Tween 80 emulsions, but the values were not significantly different (*p* > 0.05). The melting behavior of SS and SU (Figure 1) confirms the presence of crystallinity which was maximal based on the absence of crystallization peak when the emulsions were cooled directly from 37°C (data not shown). Also, statistically similar (*p* < 0.05) peak melting temperatures of 52.2 ± 0.1 and 52.0 ± 0.1°C were observed for SS and SU, respectively. DSC also confirmed the absence of crystallinity in LS and LU as no melting events were observed.

**TABLE 1:** Fatty acid composition of bulk lipids and emulsifiers.^1^

FA	Palm stearin (wt%)	Palm olein (wt%)	Tween 80 (wt%)	Span 60 (wt%)
C14:0	1.1 ± 0.0	0.8 ± 0.0	0.0 ± 0.0	0.5 ± 0.0
C16:0	58.7 ± 0.0	36.4 ± 0.1	0.9 ± 0.0	49.7 ± 0.1
C18:0	4.9 ± 0.0	4.1 ± 0.0	2.9 ± 0.0	49.2 ± 0.1
C18:1c9	26.8 ± 0.1	45.2 ± 0.0	92.1 ± 0.1	0.0 ± 0.0
C18:1c11	0.7 ± 0.1	1.4 ± 0.1	0.0 ± 0.0	0.0 ± 0.0
C18:2n6	6.2 ± 0.0	10.4 ± 0.0	0.3 ± 0.0	0.0 ± 0.0
C18:4n3	0.0 ± 0.0	0.0 ± 0.0	1.4 ± 0.0	0.0 ± 0.0
C20:0	0.4 ± 0.0	0.4 ± 0.0	1.0 ± 0.0	0.4 ± 0.0
Total	98.6 ± 0.0	98.9 ± 0.0	98.6 ± 0.0	99.9 ± 0.0

*^1^Values are mean ± SD.*

**TABLE 2:** Composition and properties of 20% oil-in-water emulsions.^1,2^

Emulsion	Lipid	Emulsifier	SFC at 37°C (%)	D_4,3_ (μm)	D_3,2_ (μm)	ζ -Potential (mV)
SS	Palm stearin	Tween 80 (2.2%)	8.78 ± 0.29^a^	0.17 ± 0.00^a^	0.12 ± 0.00^a^	−36.06 ± 6.57^a^
SU	Palm stearin	Span 60 (2.5%)	9.75 ± 0.22^a^	0.22 ± 0.01^b^	0.13 ± 0.00^b^	−42.12 ± 7.42^b^
LS	Palm olein	Tween 80 (2.2%)	0.66 ± 0.22^b^	0.21 ± 0.00^c^	0.13 ± 0.00^c^	−33.57 ± 4.07^a^
LU	Palm olein	Span 60 (2.5%)	1.9 ± 0.27^b^	0.23 ± 0.00^d^	0.14 ± 0.00^d^	−43.21 ± 7.3^b^

*^1^Values with different letters in each column are significantly different (p < 0.05). ^2^Values are mean ± SD.*

**FIGURE 2:**
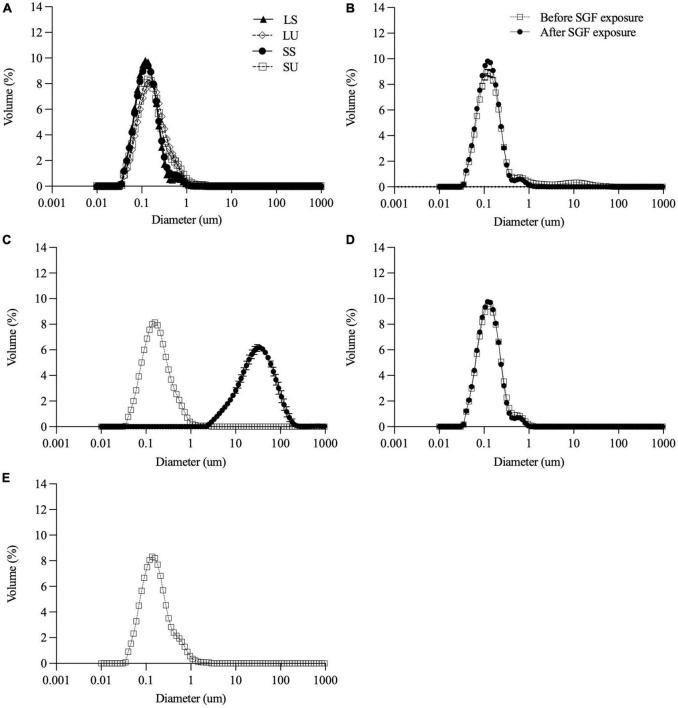
Particle size distribution of all emulsions **(A)** before digestion and of LS **(B)**, LU **(C)**, and SS **(D)** before and after 2-h exposure to SGF. Particle size distribution before digestion only for SU **(E)** since measurement after digestion was not feasible due to extensive destabilization. Data reported as mean ± SEM, *n* = 3. LS, liquid lipid-acid stable emulsifier; LU, liquid lipid-acid unstable emulsifier; SS, solid lipid-acid stable emulsifier; SU, solid lipid-acid unstable emulsifier; SGF, simulated gastric fluid.

**FIGURE 3:**
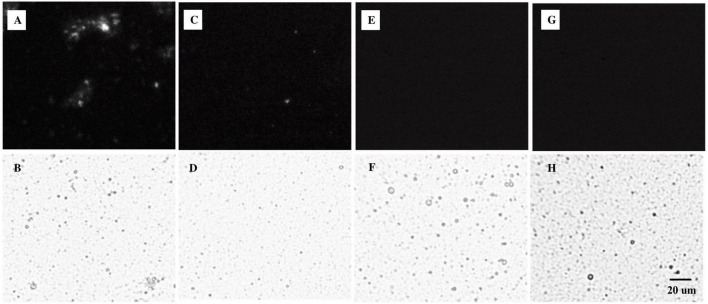
Microstructures of **(A,B)** SS, **(C,D)** SU, **(E,F)** LS, and **(G,H)** LU emulsion droplets under **(A,C,E,G)** polarized light and **(B,D,F,H)** bright-field conditions. LS, liquid lipid-acid stable emulsifier; LU, liquid lipid-acid unstable emulsifier; SS, solid lipid-acid stable emulsifier; SU, solid lipid-acid unstable emulsifier.

All emulsions had monomodal overlapping PSDs before digestion (Figure 2). Minor differences in the volume-weighted (D_4,3_) and surface area-weighted (D_3,2_) mean diameters (Table 2) were present. ζ-Potential values (Table 2) indicate that both non-ionic emulsifiers had highly negative charged droplets that differed between (*p* > 0.05), but not within each emulsifier. The Span 60 emulsion (∼−43 mV) charge was close to ∼−56 mV as reported previously for 10% palm stearin emulsions with 0.4% Span 60 (19). Similar ζ-potentials (i.e., ∼−38 versus −35 mV) have been reported for 10% soybean and Tween 80 emulsions where there was no difference due to emulsifier concentration (i.e., 0.2, 0.6, and 1%) (41). Droplet morphology imaged by bright-field light microscopy indicated that all emulsions had dispersed, spherical droplets of similarly small size (Figures 3B,D,F,H). Under cross-polarized light, only SS and SU exhibited birefringence corresponding to the presence of crystalline TAGs (Figures 3A,C) which were absent in LS and LU (Figures 3E,G).

### Exposure to Simulated Gastric Fluid

**FIGURE 4:**
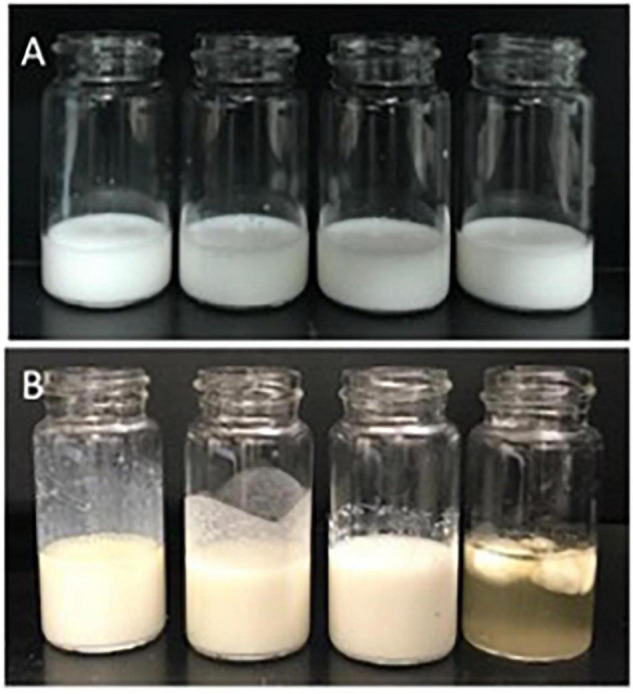
Emulsion appearance before **(A)** and after **(B)** 2-h exposure to SGF. From left to right: LS, LU, SS, and SU. LS, liquid lipid-acid stable emulsifier; LU, liquid lipid-acid unstable emulsifier; SS, solid lipid-acid stable emulsifier; SU, solid lipid-acid unstable emulsifier; SGF, simulated gastric fluid.

Gastric stability was determined visually (Figure 4) and by measuring particle size (Figure 2B–E) before and after 2 h SGF static *in vitro* digestion exposure. As intended, LS and SS did not visibly flocculate or coalesce (Figure 4), nor did shifts occur in the PSD. There were no visual changes in LU observed with SGF exposure (Figure 4), but droplet size increased, with the monomodal peak shifting to the right (Figure 2C). SU visibly destabilized, coalescing into large phase-separated aggregates (Figure 4), and preventing representative sampling for PSD measurements.

**FIGURE 5:**
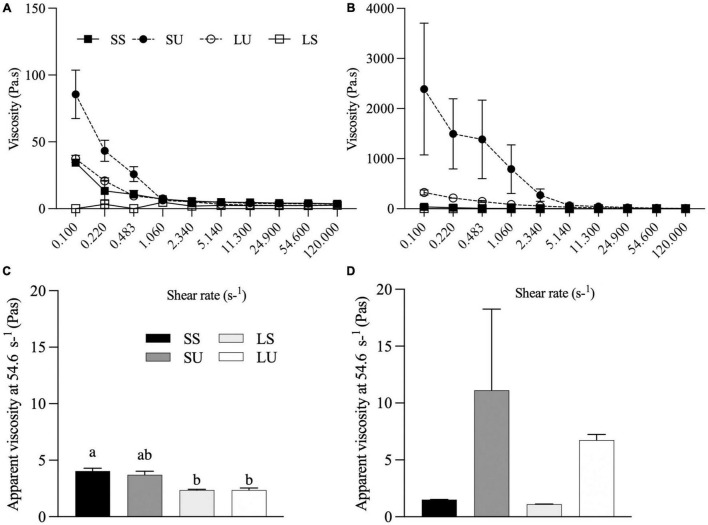
Flow behavior of emulsions before **(A)** and after **(B)** 1 h of gastric digestion. Apparent viscosity (Pa•s) at 56.23 of emulsions before **(C)** and after **(D)** 1 h of gastric digestion (*n* ≥ 2). Data reported as mean ± SEM. Bars with different letters are significantly different (*p* > 0.05). LS, liquid lipid-acid stable emulsifier; LU, liquid lipid-acid unstable emulsifier; SS, solid lipid-acid stable emulsifier; SU, solid lipid-acid unstable emulsifier.

The viscoelastic properties of each emulsion determined before and after 1 h of static gastric digestion (Figure 5) reveal shear-thinning, most obviously for SU and SS and LU both before and after digestion. In contrast, the LS emulsion and LS gastric digestate have Newtonian flow profiles (Figures 5A,B). Values of apparent viscosity at a shear rate of 56.23 s^–1^ were compared since this is within the range of physiological relevance in the GI tract (42). SS had a significantly higher apparent viscosity compared to LS (*p* = 0.038) and LU (*p* = 0.037) before digestion (Figure 4A). No significant differences were observed in the digestate’s apparent viscosity at 56.23 s^–1^ after 1-h digestion, although non-significant increases occurred for SU and LU, consistent with the anticipated flocculation of these Span-stabilized emulsions at acidic pH (Figures 5C,D).

### TIM-1 Free Fatty Acids

**FIGURE 6:**
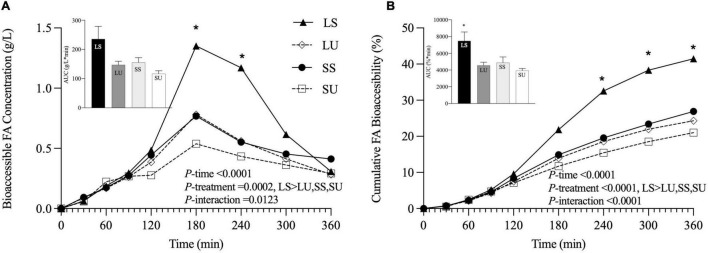
Line graphs representing bioaccessible FA concentration (g/L) **(A)** and cumulative FA bioaccessibility (%) **(B)** over time, with inset bar graphs showing AUC of LS, LU, SS, and SU during 6-h *in vitro* TIM-1 digestion (*n* ≥ 3). Data reported as mean ± SEM. Asterisk indicates a significant difference between LS and all other emulsions, *p* < 0.05. LS, liquid lipid-acid stable emulsifier; LU, liquid lipid-acid unstable emulsifier; SS, solid lipid-acid stable emulsifier; SU, solid lipid-acid unstable emulsifier; FA, fatty acid; AUC, area under the curve.

The absolute and cumulative FA bioaccessibility (calculated based on the jejunal and ileal dialysate samples) for each emulsion was collected over the 6-h TIM-1 digestion (Figure 6). According to the RM-ANOVA, LS has a significantly higher overall bioaccessible FA concentration than LU (*p* = 0.005), SS (*p* = 0.023), and SU (*p* = 0.0003). LS also had a significantly higher overall bioaccessibility than LU (*p* < 0.0001), SS (*p* < 0.0001), and SU (*p* < 0.0001). Differences were specifically observed for LS compared to all other treatments at 180, 240, and 300 min, based on FFA concentration (Figure 6A), and at 180, 240, 300, and 360 min (*p* < 0.05), based on cumulative FA bioaccessibility (Figure 6B).

**FIGURE 7:**
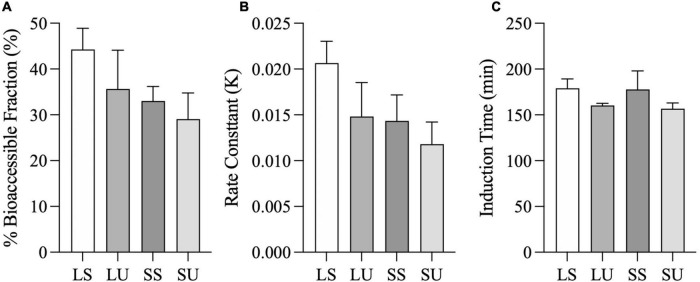
Total fatty acid bioaccessible fraction (i.e., C_*asymp*_) **(A)**, rate constant (i.e., K) **(B)**, and induction time (i.e., t_*c*_) **(C)**, calculated from the shifted logistic model for the combined jejunal and ileal filtrates. Error bars represent SEM for three replicates. No significant differences between samples were observed (*p* > 0.05).

Area under the curve values of the FFA concentration data were not significantly different (*p* = 0.07), although LS was at least 52% higher than all other emulsions. AUC values of the cumulative bioaccessibility results were significantly different (*p* = 0.03), but there were no pairwise differences based on *post-hoc* testing (*p* > 0.05). There were neither differences in terms of bioaccessible fraction nor in the rate constant values obtained from the shifted logistic model (Figure 7) (*p* < 0.05). At 6 h, LS had at least 24 and 39% higher bioaccessible fraction and rate constants (*k*), compared to all other emulsions. Similar induction times were observed (*p* > 0.05) due to the TIM-1 pre-set GE rate. Regardless of the analysis method, there were no significant differences in FA concentration, AUC, or time point differences among LU, SS, and SU (*p* > 0.05). Postprandial TAG values did not return to baseline by 6 h for two of the emulsions (LS and LU), and lipolysis was incomplete after 6-h TIM-1 for all of the emulsions (Figure 6A).

### Correlation Between TIM-1 and Human Data

**FIGURE 8:**
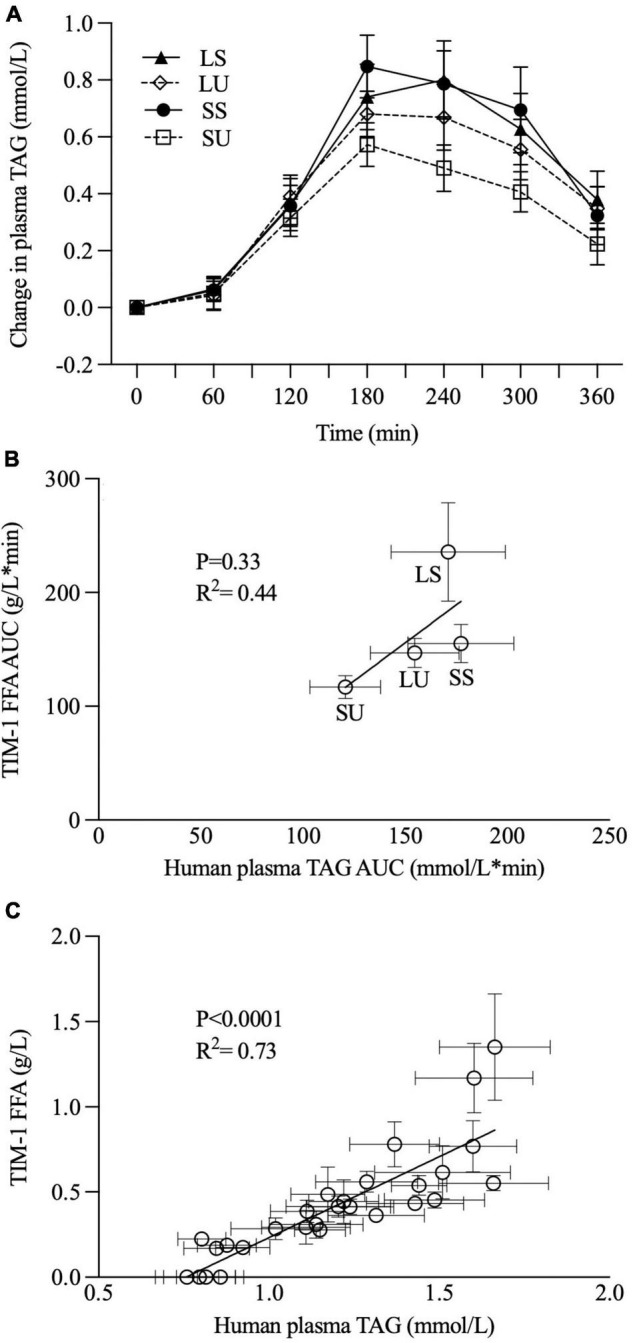
Postprandial changes in plasma TAG concentration were observed when healthy men consumed 250 ml of SS, SU, LS, and LU **(A)**, as previously reported [Hamad et al., (29)] and correlation analyses between TIM-1 6-h bioaccessible FFA AUC (g/L*min, *n* ≥ 3) and human study 6-h plasma TAG AUC (mmol/L*min, *n* = 15) **(B)** and between TIM-1 bioaccessible FFA (g/L, *n* ≥ 3) and human study plasma TAG data over 6 h **(C)**. Error bars represent SEM. LS, liquid lipid-acid stable emulsifier; LU, liquid lipid-acid unstable emulsifier; SS, solid lipid-acid stable emulsifier; SU, solid lipid-acid unstable emulsifier; TAG, triacylglycerol; FFA, free fatty acid; AUC, area under the curve.

The TIM-1 FFA values were positively correlated with postprandial TAG concentration (*p* < 0.0001, *R*^2^ = 0.74) (Figure 8C), but the overall TIM-1 and human study trends did not align (Figures 8A,B).

### Adjusted Gastric Emptying TIM-1 Free Fatty Acids

**FIGURE 9:**
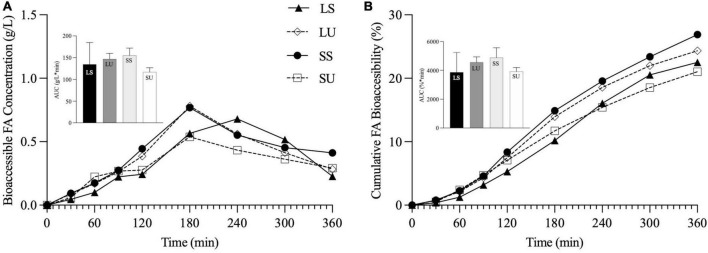
Line graphs representing bioaccessible FA concentration (g/L) **(A)** and cumulative FA bioaccessibility (%) **(B)** over time, and inset bar graphs representing AUC of LS, LU, SS, and SU during 6-h in vitro TIM-1 digestion with modified gastric emptying half time for LS (*n* ≥ 3). Data reported as mean ± SEM. LS, liquid lipid-acid stable emulsifier; LU, liquid lipid-acid unstable emulsifier; SS, solid lipid-acid stable emulsifier; SU, solid lipid-acid unstable emulsifier; TAG, triacylglycerol; FA, fatty acid; AUC, area under the curve.

TIM-1 GE *T*_1/2_ was set to 124 min for LS and was kept at the default 80 min for SU. This difference was intentionally proportional to the observed longer GE *in vivo* for LS versus SU (29). Figure 9A shows the bioaccessibility data with this adjustment in GE. According to Figure 9B, TIM-1 FA cumulative bioaccessibility and AUC values for LS were not significantly different from all other treatments. With this adjustment in GE, TIM-1 FA cumulative bioaccessibility and AUC values for LS were not significantly different from all other treatments (Figure 9B).

## Discussion

This study aimed to determine the *in vitro* lipolysis and FA bioaccessibility of four lipid emulsions formulated to contain partially solid or liquid oil droplets at 37°C, and to either be susceptible to flocculation or remain dispersed in the acidic environment of the stomach. This is one of the first studies to control for the individual and combined effects of emulsion TAG crystallinity and acidic colloidal stability. The use of the TIM-1 digestion model offered a rare opportunity to directly compare the bioaccessibility findings from this leading digestion simulator with PPL results from a human study in which 15 healthy men consumed the same test emulsions, namely SS, SU, LS, and LU (29), and to explore strategies to modify the *in vitro* digestion protocol toward the aim of physiological relevance.

### TIM-1 Free Fatty Acids Bioaccessibility

#### Differences Based on Triacylglycerol Crystallinity

The above results support that emulsion droplet crystallinity attenuates *in vitro* intestinal lipolysis, evidenced by the lower lipolysis of the two solid lipid emulsions (SU and SS) compared to LS. The lower bioaccessibility for SS compared to LS, specifically, provides the strongest indication that SFC was predominantly responsible for the differences since these emulsions were both stable during gastric digestion. Similar observations have been reported showing that acid-stable emulsions formulated with canola oil had higher TAG hydrolysis compared with those containing crystalline TAG (based on canola stearin, SFC ∼12.4%) (15). For example, Guo et al. observed lower lipolysis for emulsions that contained crystalline TAG (13). In that case, the lipolysis rate was proportional to blend SFC at 37°C, with the highest and lowest rates observed for emulsions containing the liquid oil and hardstock, respectively (13). Similarly, Day et al. observed higher *in vitro* lipolysis with emulsions containing liquid canola oil stabilized with both 1% sodium caseinate and 0.25% monoglyceride compared to an emulsion containing partially crystalline TAG (6% hydrogenated vegetable oil and 14% canola oil) (43). In the same report, a significant 2-h increase in human whole blood TAG was observed for the liquid state system, compared with a more modest, non-significant increase for the emulsion with crystalline TAG. In contrast, Wan et al. recently reported that 20% palm stearin versus palm olein emulsions stabilized with sodium caseinate had a significantly greater extent of FA release at the end of a 2-h static *in vitro* digestion (44). According to the authors, the enhanced lipolysis for PS was attributed to the penetration of solid fat crystals at the oil–water interface, allowing greater lipase access and easier removal of digestion products (44). However, this study also with palm-based lipids and using a relatively more complex *in vitro* digestion method supports the conclusion that emulsion TAG crystallinity generally has an attenuating influence on digestibility.

#### Differences Based on Emulsifier Acid Stability

Another important bioaccessibility observation is that emulsion acid stability impacted digestibility in the absence of TAG crystallinity. This was evidenced by the higher bioaccessibility for LS compared to LU which we speculate is attributed to LS’s higher interfacial area upon reaching the duodenal phase and lower digestate viscosity. The finding agrees with a human study where an acid-stable emulsion showed higher postprandial-labeled lipid chylomicrons compared to acid-unstable emulsions (25). In the current work, exposure to SGF led to increases in particle size (but not phase separation) for LU, while LS was unchanged. Therefore, LU is expected to have relatively larger particles and a correspondingly lower interfacial area for lipolysis when it enters the duodenal compartment (12). *In vitro*, (45) *in vivo* animal, (46) and human (47) studies confirm that a smaller particle size is correlated with higher lipolysis.

Beyond particle size, the composition and nature of the interface affect the ease of lipase binding and hydrolysis (12); thus, Span 60 and Tween 80 may affect lipase activity differently. Although palm olein is completely a liquid at body temperature (i.e., melting point ∼28°C) (48), small amounts of crystallinity were observed for LS and LU, related to the emulsifier FA tails at the interface. Tween 80 contains predominantly oleic acid, while Span 60 contains equal proportions of palmitic and stearic acids which may interact differently with the droplet TAG. We previously observed the presence of a crystalline shell for Span 60 palm stearin and canola oil emulsions (19), which may contribute to the differences in lipase binding and microstructural changes during digestion. Otherwise, LU and LS possessed similar properties initially, yet TIM-1 FA bioaccessibility was greater for LS than LU, supporting the conclusion that, in the absence of TAG crystallinity, resistance to acidic flocculation led to enhanced FA bioaccessibility.

#### Differences Based on the Combined Effects of Triacylglycerol Crystallinity and Emulsifier Acid Stability

It is not surprising that different lipolysis trends are observed for liquid versus solid TAGs depending on the emulsifier present. The differences in digestibility between liquid- and solid-state droplets are attributed to restricted lipase access when less mobile crystalline TAG is present at an emulsion interface (15). However, direct evidence of these differences is rare, given that flocculation and partial coalescence can occur in the presence of solid fat. For example, within the acid-unstable emulsions (LU and SU), bioaccessibility over time did not differ significantly, although LU had a 15% higher cumulative FFA release at the end of the 6-h digestion compared to SU. In the previous studies, liquid oil-in-water emulsions with acid-unstable emulsifiers were subsequently redispersed, while those containing crystalline TAG did not (19, 28). In this study, SU and LU (20) were susceptible to acid-induced colloidal changes, and when the digestate pH was raised to 7 at the start of the duodenal phase, partial redispersal occurred for LU, but SU did not redisperse (data not shown). Comparing SS (resisted acidic flocculation) and SU (partial coalescence and phase separation in the gastric phase) indicates minimal to no differences in droplet digestibility based on TAG crystallinity. This is somewhat surprising since SU presumably had a lower interfacial area available for lipolysis, but conceivably this factor is less significant for crystalline TAG.

Overall, based on the TIM-1 results, both TAG crystallinity (LS > SS) and acid instability (LS > LU) have the potential to attenuate lipolysis independently. Nonetheless, the combination of crystalline TAGs and acid-unstable emulsifier (SU) resulted in the greatest gastric instability (i.e., complete separation of the water and lipid phases into clumps which resisted redispersion) and the trend toward the lowest bioaccessibility compared to all other emulsions.

### TIM-1 and Human Data Comparison

The positive correlation between human postprandial TAG concentration and the TIM-1 FFA values is not surprising given that both TIM-1 FFA values are strongly time-dependent. However, it is very important that the overall trends from the TIM-1 and human studies point to different conclusions about the role of TAG crystallinity and emulsion acid stability. *In vivo*, no significant differences were observed in TAG iAUC (as an indicator of overall TAG absorption), but TIM-1 bioaccessibility was higher for LS. The trends in TIM-1 AUC values based on FFA concentration also do not agree with those based on the human postprandial TAG AUC. Therefore, the human study results do not concur with the observed higher TIM-1 bioaccessibility for LS. However, both *in vitro* and *in vivo* models suggest that acidic flocculation in the presence of TAG crystallinity has an attenuating influence. The TIM-1 FFA AUC value for SU was 50, 26, and 20% lower than LS, SS, and LU, respectively, but only LS was statistically different from the other treatments. As previously reported, human PPL AUC TAG data for SU trended to be 29, 32, and 22% lower compared to LS, SS, and LU, respectively, with no appreciable differences between LS and SS or SU (29). These PPL differences are of a similar magnitude as where statistical significance was previously observed using a very similar human study design (20), pointing to implications of nuanced differences in emulsion physical properties for PPL. This is especially exciting given the wide-ranging implication PPL has for metabolism, e.g., CVD risk, satiety, inflammation related to lipopolysaccharide absorption, beta-oxidation, and interactions with gut microbiota.

There are many reasons why the results from *in vitro* and *in vivo* methods may not align precisely. In this case, differences in GI transit may help to explain the apparent discrepancies. GE is controlled by a complex feedback mechanism *via* gastrointestinal hormones (49), which, along with antral contraction, can be influenced by food properties (37). GE is a major factor that controls the rate of lipolysis *in vivo* (14). In the human study, ultrasonography confirmed the differences in gastric structure and GE rate between the emulsions (29). Thus, we exploited this in vivo data to explore whether adjusting the TIM-1 GE rate would achieve better alignment between the *in vitro* and *in vivo* findings.

### TIM-1 Free Fatty Acids Bioaccessibility With Adjusted Gastric Emptying

According to ultrasound imaging, the acid-stable emulsions (LS and SS) remained dispersed in the stomach, as intended, and had significantly faster GE than SU and LU, which extensively flocculated and phase-separated in the stomach (29). *In vivo* GE half times were significantly higher for the acid-stable emulsions (∼300 and 262 min for LS and SS versus ∼184 and 190 min for LU and SU) (29). As above, the TIM-1 applies a pre-set GE rate based on a half time of 80 min (TNO, Zeist, Netherlands) (50) and lacks feedback mechanisms to adjust motility and meal transit. As such, the TIM-1 experiments may have limited ability to detect digestibility differences that occur *in vivo* based on differences in gastric structuring and subsequent GE. To explore this, a second set of TIM-1 experiments was conducted, taking into consideration the *in vivo* GE rate differences. When the TIM-1 GE *T*_1/2_ was adjusted for LS, its results no longer differed from the other three emulsions, and the results for SU remained lower than all other emulsions as observed *in vivo*. This confirms that the GE rate is a plausible main explanation for the observed lack of precise alignment between the *in vitro* TIM-1 and *in vivo* human results. This finding urges caution in comparing the results for meals where gastric structures and emptying are expected to differ. There are reports of an agreement between TIM-1 bioaccessibility and postprandial TAG response (35, 36, 51), and differences in the alignment of *in vitro* and TIM-1 findings may relate to differences in the form of lipids being tested (i.e., emulsified or not and SFC) and the degree of associated structural changes in the stomach. In this case, when GE differences were accounted for, TIM-1 bioaccessibility more closely reflected the outcomes observed *in vivo*. Effectively, this refinement in *in vitro* transit corrected for an apparent major augmentation of lipid bioaccessibility for LS. *In vivo*, the lipid phase of the LS (and SS) emulsions emptied the stomach earlier than the separated lipids from SU and LU, triggering the ileal brake which led to a decrease in gastric motility (29), altering lipid absorption kinetics in a way that the TIM-1 cannot mimic. Incidentally, although PPL did not differ significantly between the emulsions (*p* > 0.05), substantial differences were observed in satiety hormones and participant ratings.

The improved *in vitro–in vivo* correlation with adjusted GE half times highlights the utility of non-invasive ultrasonography to obtain physiologically relevant GE rates to improve the predictability of *in vitro* digestion models. Other reasons *in vitro* results may not mirror human findings, which arise due to various pre- and post-absorptive processes (52). For example, while TIM-1 fractionates the absorbable (i.e., bioaccessible) lipids from the digestate, it cannot account for potential systemic differences in *in vivo* clearance of absorbed TAG. Moreover, both the *in vitro* and the *in vivo* results point to the need for longer experimental protocol postprandial TAG values since the two liquid emulsions had not returned to baseline by 6 h, and TIM-1 lipolysis was incomplete for all of the emulsions at that timepoint.

## Conclusion

This study investigated the individual and combined effects of TAG physical state and colloidal acid stability of palm-based oil-in-water emulsions on *in vitro* lipid bioaccessibility using a computer-simulated dynamic digestion model TIM-1. The findings were compared to an acute postprandial TAG study in which healthy men consumed the same emulsions. Overall, the combination of an acid-stable emulsifier and liquid oil (i.e., the LS emulsion) led to the highest TIM-1 digestibility, although there were no differences in the shifted logistic model parameters. The trends observed based on AUC values from the *in vitro* and *in vivo* methods did not concur. Specifically, TIM-1 did not indicate the same treatment differences observed *in vivo.* It was hypothesized that this was because of *in vivo* GE differences between the emulsions. Indeed, when the TIM-1 GE rate was adjusted to reflect the differences in emptying observed *in vivo*, the *in vitro* TIM-1 FFA response more closely resembled the human lipemic response, with the combination of solid lipid and acid-unstable emulsifier leading to the lowest digestibility both *in vivo* and *in vitro*. This provides insight into the importance of considering gastric meal structures and corresponding differences in GE in future *in vitro* digestion studies. Overall, emulsion TAG properties can be tailored for different gastrointestinal behaviors and *in vitro* and *in vivo* methods should be integrated for a comprehensive understanding of how food properties influence metabolic response.

## Data Availability Statement

The raw data supporting the conclusions of this article will be made available by the authors, without undue reservation.

## Ethics Statement

The studies involving human participants were reviewed and approved by the University of Guelph Human Research Ethics Board. The participants provided their written informed consent to participate in this study.

## Author Contributions

SH and AW designed the research and analyzed the data. SH, RC, YL, NR, ZZ, and PN conducted the research. SH, MR, and AW interpreted the data and wrote the manuscript. All authors read and approved the final manuscript.

## Conflict of Interest

The authors declare that the research was conducted in the absence of any commercial or financial relationships that could be construed as a potential conflict of interest.

## Publisher’s Note

All claims expressed in this article are solely those of the authors and do not necessarily represent those of their affiliated organizations, or those of the publisher, the editors and the reviewers. Any product that may be evaluated in this article, or claim that may be made by its manufacturer, is not guaranteed or endorsed by the publisher.

## Abbreviations

GE: gastric emptying
PPL: postprandial lipemia
SFC: solid fat content
GI: gastrointestinal
LS: liquid lipid-acid stable emulsifier
LU: liquid lipid-acid unstable emulsifier
SS: solid lipid-acid stable emulsifier
SU: solid lipid-acid unstable emulsifier
TAG: triacylglycerol
FA: fatty acid
FFA: free fatty acid
AUC: area under the curve.
